# Supplementary material for: The Development of New Methods to Stimulate the Production of Antimicrobial Peptides in the Larvae of the Black Soldier Fly Hermetia illucens
Source: Int J Mol Sci. 2023 Oct 30;24(21):15765. doi: 10.3390/ijms242115765 (PMC10647447; doi:10.3390/ijms242115765)
Supplement: Supplementary file 1 [file ijms-24-15765-s001.zip › ijms-2677030-supplementary.pdf]

The development of new methods to stimulate the production of antimicrobial peptides in the larvae of the black soldier fly *Hermetia illucens*

Atsuyoshi Nakagawa[1,2], Takuma Sakamoto[3], Michael R. Kanost[4], and Hiroko Tabunoki[2,3]*

[1]Division of Research & Development, Chiba Research Laboratory, UBE Corporation, 8-1 Goi-Minamikaigan, Ichihara, Chiba 290-0045, Japan

[2]Cooperative Major in Advanced Health Science, Graduate School of Bio-Applications and System Engineering, Tokyo University of Agriculture and Technology, Tokyo, Fuchu, 183-8509, Japan

[3]Department of Science of Biological Production, Graduate School of Agriculture, Tokyo University of Agriculture and Technology, 3-5-8 Saiwai-Cho, Fuchu, Tokyo 183-8509, Japan

[4]Department of Biochemistry and Molecular Biophysics, Kansas State University, 141 Chalmers Hall, Manhattan, KS 66506-3702, USA

*To whom correspondence should be addressed. E-mail: h_tabuno@cc.tuat.ac.jp

Supplementary information

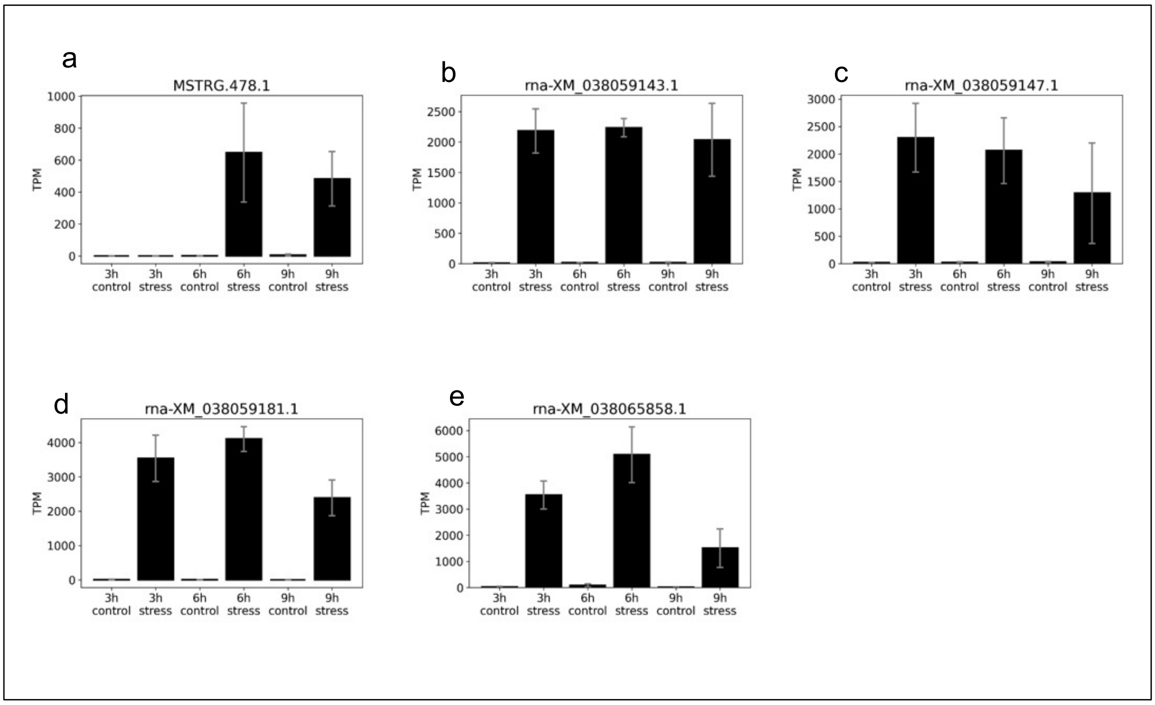

Figure S1 The expression of the transcripts annotated to Defensin (NM_078948.3). The y-axis indicates the ratio of the average Transcripts Per Kilobase Million (TPM) values for each transcript between the control and thermal injury groups.

a, transcript for MSTRG.478.1; b, transcript for rna-XM_038059143.1;c, transcript for rna-XM_038059147.1;d, transcript for rna-XM_038059181.1;e, transcript for rna-XM_038065858.1.

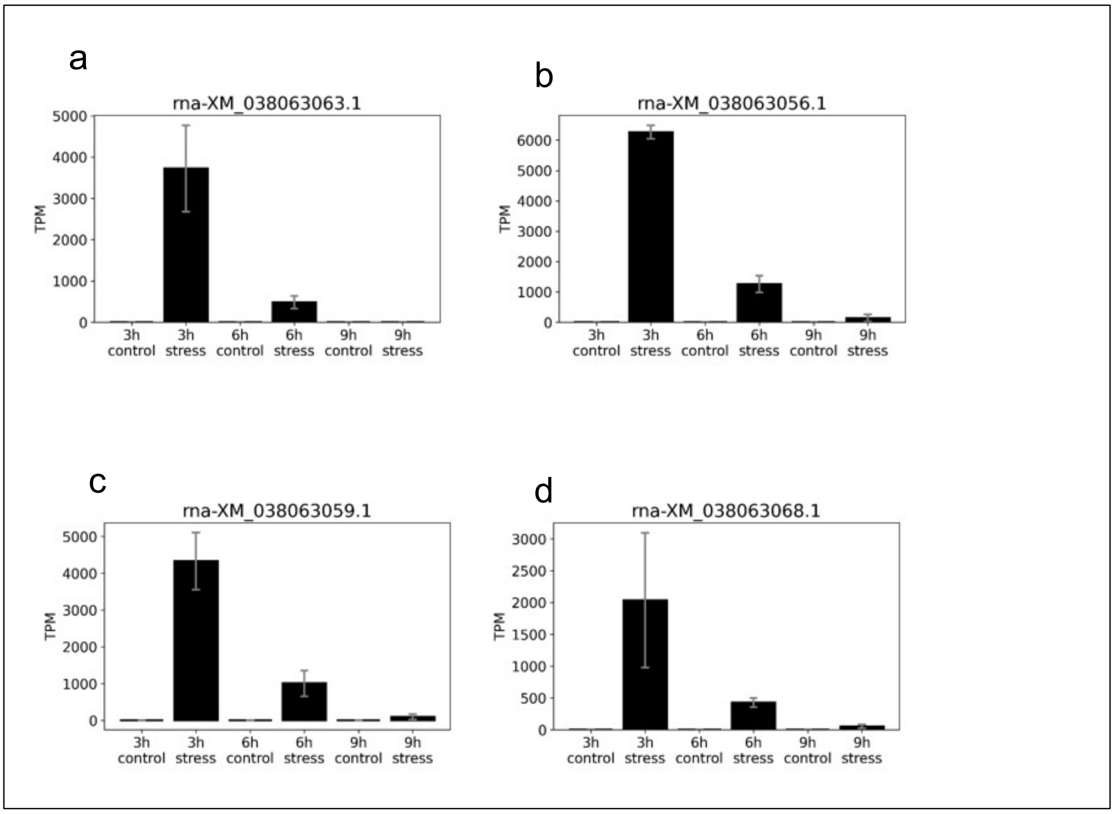

Figure S2 The expression of the transcripts annotated to Cecropin A2 (NM_079850.4). The y-axis indicates the ratio of the average Transcripts Per Kilobase Million (TPM) values for each transcript between the control and thermal injury groups.

a, transcript for rna-XM_038063063.1; b, transcript for rna-XM_038063056.1;c, transcript for rna-XM_038063059.1;d, transcript for rna-XM_038063068.1.

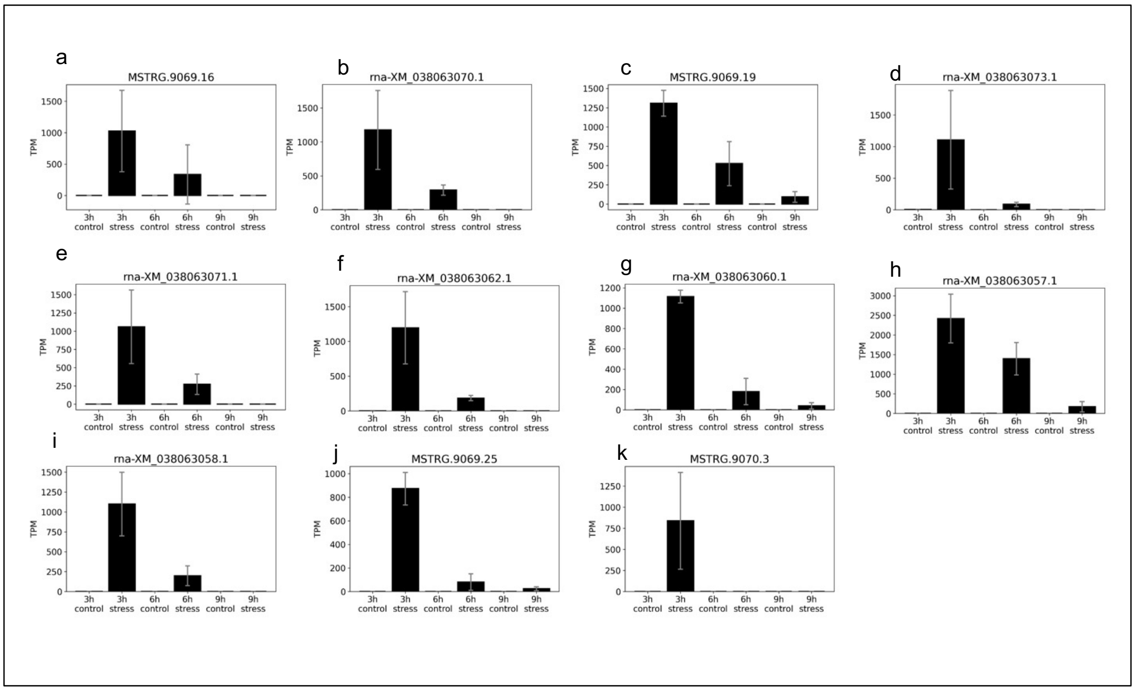

Figure S3 The expression of the transcripts annotated to Cecropin C (NM_079852.3). The y-axis indicates the ratio of the average Transcripts Per Kilobase Million (TPM) values for each transcript between the control and thermal injury groups. a, transcript for MSTRG.9069.16; b, transcript for rna-XM_038063070.1;c, transcript for MSTRG.9069.19; d, transcript for rna-XM_038063073.1.; e, transcript for rna-XM_038063071.1.; f, transcript for rna-XM_038063062.1.; g, transcript for rna-XM_038063060.1.; h, transcript for rna-XM_038063057.1.; i, transcript for rna-XM_038063058.1.; j, transcript for MSTRG.9069.25; k, transcript for MSTRG.9070.3.

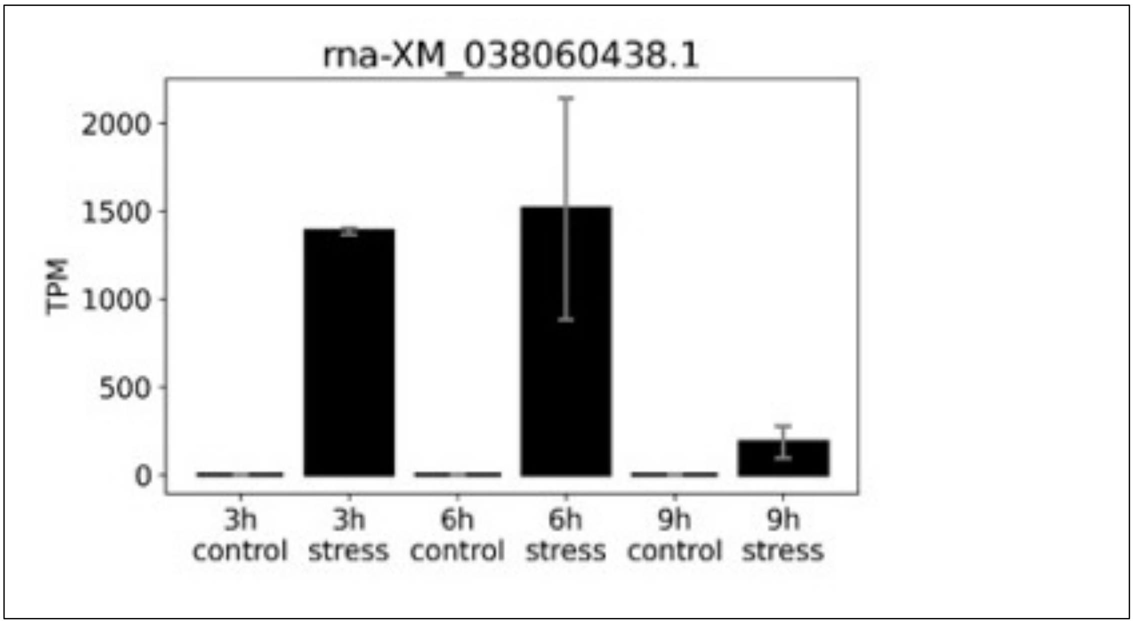

Figure S4 The expression of the transcripts annotated to Attacin-A (NM_079021.5). The y-axis indicates the ratio of the average Transcripts Per Kilobase Million (TPM) values for the transcript between the control and thermal injury groups.

The graph indicates the transcript for rna-XM_038060438.1.

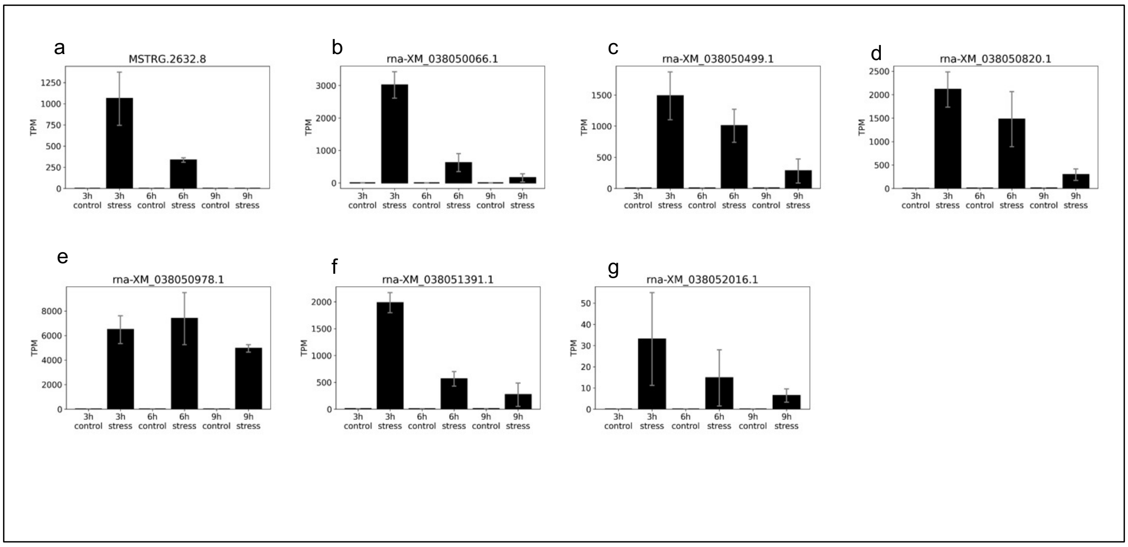

Figure S5 The expression of the transcripts annotated to Diptericin B (NM_079063.4). The y-axis indicates the ratio of the average Transcripts Per Kilobase Million (TPM) values for each transcript between the control and thermal injury groups.

a, transcript for MSTRG.2632.8; b, transcript for rna-XM_038050066.1;c, transcript for rna-XM_038050499.1; d, transcript for rna-XM_038050820.1.; e, transcript for rna-XM_038050978.1.; f, transcript for rna-XM_038051391.1.; g, transcript for rna-XM_038052016.1.

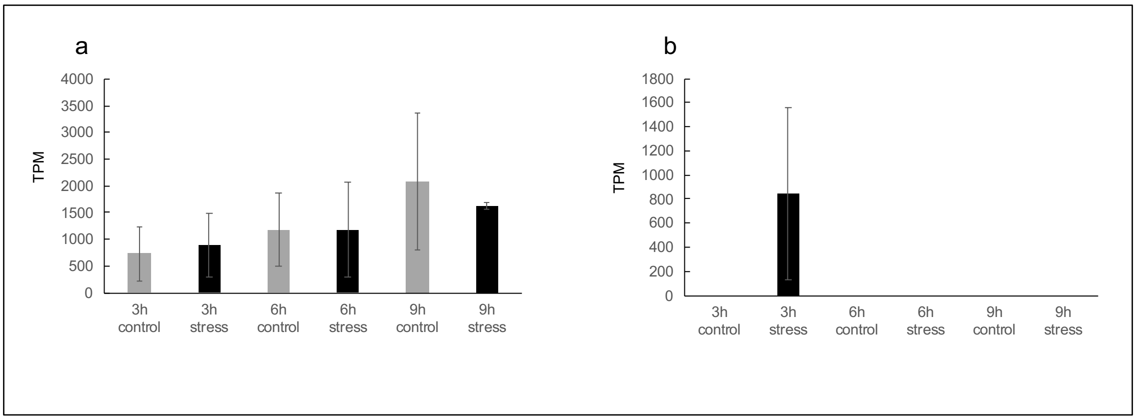

Figure S6 The expression of the transcripts annotated to phenoloxidase (XM_038046567.1). The y-axis indicates the ratio of the average Transcripts Per Kilobase Million (TPM) values for the transcript between the control and thermal injury groups. The graph indicates the transcript for XM_038046567.1

Table S1. Primers for RT-qPCR were used in this study.

Gene name	Forward (5'-3')	Reverse (5'-3')
Hirs18	CGATGGCAAGTACACACAGC	AATCCACCAACGCGACATTG
HiCLP1	CAGAACATTGGCTCCTTGCT	TTTCGTTGTTTTCGCTGTTG
HiDLP4	CATTGCCAACCTTTCCAACT	GTCACACCATCCTCCTCGTT
HiPPO	AGACTTGGAGCCGCTAATCA	GGGACCTATTTGGCTGCATA
HiPGRP-SA	CGTCCCCAATGTGATAATCC	TTATGCCAACCTACGCCTTC

Table S2. Primers for cDNA cloning were used in this study.

Gene name	Forward (5'-3')	Reverse (5'-3')
HiCLP 1	ATGAATTTCACTAAGCTTTTCGT T	TTATCCTTGTTGTGGTGGTCCA
HiDLP 4	ATGGTCCATTGCCAACCTTTCCA A	CTATTTCCGGCAGTTGCAAACA GC

Table S3. SRA accession numbers for RNA-seq data.

SRA accession numbers	Samples information
DRR426824	Control 1 for 3h
DRR426825	Control 2 for 3h
DRR426826	Control 3 for 3h
DRR426827	Thermal injury 1 for 3h
DRR426828	Thermal injury 2 for 3h
DRR426829	Thermal injury 3 for 3h
DRR426830	Control 1 for 6h
DRR426831	Control 2 for 6h
DRR426832	Control 3 for 6h
DRR426833	Thermal injury 1 for 6h
DRR426834	Thermal injury 2 for 6h
DRR426835	Thermal injury 3 for 6h
DRR426836	Control 1 for 9h
DRR426837	Control 2 for 9h
DRR426838	Control 3 for 9h
DRR426839	Thermal injury 1 for 9h
DRR426840	Thermal injury 2 for 9h

DRR426841	Thermal injury 3 for 9h

BioProject: PRJDB14676, DRA submission: DRA015412
